# Enhancing the value of death registration with verbal autopsy data: a pilot study in the Senegalese urban population in 2019

**DOI:** 10.1186/s13690-023-01067-6

**Published:** 2023-03-29

**Authors:** Khadim Niang, Atoumane Fall, Samba Ndiaye, Maguette Sarr, Khady Ba, Bruno Masquelier

**Affiliations:** 1 Department of Public Health and Social Medicine, University of Gaston Berger, Saint-Louis, Senegal; 2 Agence Nationale de la Statistique et de la Démographie (ANSD), Dakar, Senegal; 3 Center for Demographic Research, Louvain University, Louvain-la-Neuve, Belgium

**Keywords:** Verbal autopsies, Death registration, Epidemiological transition, Senegal, Dakar

## Abstract

**Background:**

There is no source of data on causes of death in Senegal that covers both community and hospital deaths. Yet the death registration system in the Dakar region is relatively complete (>80%) and could be expanded to provide information on the diseases and injuries that led to death.

**Methods:**

In this pilot study, we recorded all deaths that occurred over 2 months and were reported in the 72 civil registration offices in the Dakar region. We selected the deaths of residents of the region and administered a verbal autopsy to a relative of the deceased to identify the underlying causes of death. Causes of death were assigned using the InterVA5 model.

**Results:**

The age structure of deaths registered at the civil registry differed from that of the census, with a proportion of infant deaths about twice as high as in the census. The main causes of death were prematurity and obstetric asphyxia in newborns. Meningitis and encephalitis, severe malnutrition, and acute respiratory infections were the leading causes from 1 month to 15 years of age. Cardiovascular diseases accounted for 27% of deaths in adults aged 15-64 and 45% of deaths among adults above age 65, while neoplasms accounted for 20% and 12% of deaths in these two age groups, respectively.

**Conclusions:**

This study demonstrates that the epidemiological transition is at an advanced stage in urban areas of Dakar, and underlines the importance of conducting regular studies based on verbal autopsies of deaths reported in civil registration offices.

## Background

Most low- and middle-income countries lack comprehensive systems of civil registration and vital statistics (CRVS) with high-quality data on causes of death [1]. This data gap hinders the development of health systems because it prevents the proper identification of priorities for disease and injury control, reduces the ability to evaluate health programs, and limits the preparedness for emerging epidemics [2]. The need to expand death registration systems to allow real-time tracking of cause-specific mortality has become even more evident since the development of the COVID-19 epidemic [3, 4].

Compared to other sub-Saharan African countries, Senegal has a relatively efficient civil registration system, but this system does not yet allow for the generation of mortality statistics at the national scale. According to the 2013 census, 60.7% of deaths that had occurred in the year preceding the census had been registered in urban areas, compared to 11.9% in rural areas. Respectively 31.7% and 82.4% of deaths were not registered, while the head of the household did not know how to respond for 7.5% and 5.8% of deaths [5]. The Dakar region stands out from other parts of the country, as its registration completeness reaches 83.0% of deaths. As this region accounts for half of the Senegalese urban population and more than a fifth of the total population of the country, its CRVS system could potentially become a key data source for monitoring the burden of disease and mortality, provided that the records are digitized and organized in a central database, and that information is collected on the underlying causes of death. The Senegalese government is modernizing its CRVS system to move towards the computerization of all registration offices and the digitization of registers, with the support of several development partners such as the European Union, UNICEF, and the Global Financing Facility [6]. However, these developments should be accompanied by initiatives to improve the collection of cause-of-death data.

Senegalese law does not currently require a medical certificate of death with a precise cause of death. The main sources of information on causes of death thus come from health facility statistics, which are incomplete and not always standardized. The Ministry of Health and Social Action started implementing the district health information system (DHIS-2) in 2014 in two regions and the system was scaled up to all regions in 2016. But the cause-of-death data in DHIS-2 only included public hospitals up until 2019, and a minority of private health facilities are now included. In addition, an important proportion of deaths occur at home and are not recorded in the DHIS-2 statistics, leading to a lack of representativeness [7]. Deaths at home might be certified by a physician, but the focus is often on the type of death (natural or accidental), rarely the cause of death. The only available data source on causes of death that cover both facility-based and home-based deaths refers to rural populations monitored in Health and Demographic Surveillance Systems (HDSS) [8–10]. Comparable data are sorely lacking for urban settings.

Life expectancy has increased significantly over the last decades in Senegal, from 39.2 in 1970 to 67.9 in 2019, with a temporary stall in progress in the 1990s [11]. This improvement in life expectancy has been largely driven by gains in child survival, with under-five mortality plummeting from 288‰ in 1970 to 45‰ in 2019 [12]. Improved access to health services (including antenatal care, skilled attendance at birth and immunizations), effective control of malaria, and the expansion of maternal education seem to have contributed most to this decline [13–15]. These survival gains in childhood contrast with more modest progress at adult ages [16]. As a result, the age distribution of deaths is shifting to adulthood, a trend amplified by the reduction in fertility. According to the United Nations, 56% of deaths in Senegal over the period 1990-1995 were among children under 15 years of age, and this proportion has fallen to 31% over the period 2015-2020 [11]. The hierarchy of causes of death is also modified. According to the Global Burden of Disease Study (2019), 66.7% of deaths in Senegal were caused by communicable diseases, nutritional or neonatal disorders, and pregnancy-related complications in 1990, while 26.3% of deaths were caused by non-communicable diseases, and 5% were caused by accidents and injuries [17]. Three decades later, in 2019, the cause-of-death distribution had been radically altered; the share of deaths caused by non-communicable diseases had almost doubled to reach 47.0%, compared with 46.4% of deaths caused by communicable diseases and related conditions, and 6.5% of violent deaths. Such estimates are, however, based on complex modelling, informed by few empirical measurements of deaths and their causes [2]. Moreover, they refer to the national level, whereas the epidemiological transition is expected to follow different paths according to the environment of residence. In rural areas, HDSS already highlight the high share of non-communicable diseases among adults: in Mlomp (Casamance), they caused 50.7% of deaths among men and 48.7% of deaths among women aged 15 to 60 in the period 1985-2004 (with neoplasms accounting for 28% of adult deaths) [18]. This percentage could be higher among urban residents, who are more exposed to risk factors for non-communicable diseases, such as sedentary lifestyles, diets rich in sugar and fat, and air pollution, resulting in a higher prevalence of hypertension and obesity. For example, the proportion of women who were overweight or obese was estimated at 28.9% in the 2010-11 DHS in urban areas, compared with 13.7% in rural areas [19]. Studies conducted in Dakar on hypertension and diabetes confirm that the prevalence of these conditions is quite high (24.7% and 17.9% respectively) [20, 21].

In this context, we conducted a pilot study to evaluate whether registration offices could serve as points of contact with the deceased’s relatives in order to conduct verbal autopsies to document the main causes of death in the Dakar Region. Verbal autopsies consist of interviewing family members or caregivers of the deceased using a structured questionnaire to determine the symptoms of the disease that preceded the death and the circumstances of the death to establish a probable cause [22]. This is a cost-effective and internationally comparable tool for obtaining a reasonable estimate of the distribution of causes of death at the community or population level, although it does not necessarily provide reliable estimates at the individual level [23]. In Senegal, verbal autopsies have long been used in the rural HDSS of Bandafassi, Mlomp and Niakhar [8–10, 24, 25]. But until now, the integration of verbal autopsies with the routine civil registration system had not been tested. We screened all deaths notified in the civil registration offices between December 2018 and February 2019 and collected verbal autopsies among relatives of the deceased who had lived in the region.

## Methods

### Study site

The Dakar Region is ahead of all other regions of the country in terms of economic and health infrastructure. Since June 1958, the region has become the seat of government and the capital of the country. It is the main centre of industry, commerce and finance. The population of Dakar enumerated in 2013 was 3,137,196 inhabitants, representing nearly a quarter of the population of Senegal (23.2%) on only 0.28% of the national territory. The population of the region is still very young, with 44.5% under 20 years of age [26]. As much as 50% of monetary income in Senegal is concentrated in Dakar [27]. 99.6% of households in the Dakar Region have access to an improved water source, and 96.6% have access to electricity, as compared to 85.1% and 63.3% at the national level [28]. The region hosts 11 of the 12 level-3 public health establishments of the country (at the top of the health pyramid) and is the only region to have all the medical specialties offered [29]. In 2014, the region had a regional supply pharmacy, a regional hygiene brigade, 22 health centers organized in 10 health districts, and 155 health posts [29]. The private sector is also well represented, and plays an important role in the health system of the region.

Death registration is compulsory and free in Senegal and should occur within one month. Declarations may be made by one of the parents of the deceased or by any other person who has the necessary information on his or her civil status to establish the record. Late declarations are possible after 6 weeks, but within one year after the death, provided that the relative notifying the death produces a certificate from a doctor in support of his declaration or that he/she is joined by two witnesses.

The completeness of registration, assessed directly based on the 2013 census, is around 83% in the Dakar Region [5]. An analysis of the census data revealed that deaths of adults were the most likely to be registered (respectively 84% in adults aged 15-59 and 87% in adults aged 60+, compared to 71% of deaths of children under 5 years of age). Death registration was also more frequent in households of the richest wealth quintile (90% against 77% in the poorest quintile), and when the household head was older, when he or she had attended school, and whe he or she had a birth certificate [30]. Completeness was highest in the department of Dakar (88%), followed by the departments of Guediawaye (87%), Pikine (81%) and Rufisque (76%).

### Study design

The verbal autopsy study was organized in households of relatives of the deceased, and these relatives were recruited when notifying deaths to registration offices. All 72 registration offices in the Dakar region were invited to be part of the study. All deaths that occurred in these offices were screened for eligibility. Deaths were eligible for a follow-up verbal autopsy when (1) the deceased had died in the Dakar region, (2) he or she had resided in this region for at least one month prior to death (or since birth for newborns), and (3) he or she had at least one close relative who could be reached for an interview in the Dakar region. Stillbirths were excluded from the sample. Deceased whose relatives refused to participate in the survey, were unreachable by telephone during the collection period, or were unavailable during the collection period were also not included. Due to budgetary limitations, the collection period was limited to two months. The study sample, therefore, consists of all deaths that occurred in the Dakar region between December 20, 2018, and February 20, 2019.

A screening form was first filled in by agents of the registration office receiving the relatives of the deceased to collect data on the sex and age of the deceased, check the conformity of the selection criteria, and collect the consent of the relative to set up an appointment. In the case of non-inclusion, this form was used to provide information on the causes or circumstances of refusal and the region of the deceased. In the days following the mourning period, on the basis of an appointment set by the relative of the deceased who had agreed to participate in the study, an investigator was dispatched to her home to administer the questionnaire for the verbal autopsy. The interview was conducted at the respondent’s home in a quiet room to ensure confidentiality. To conduct the interviews, 24 final-year medical students were trained in the administration of the verbal autopsy and deployed in the field. These interviewers were organized into six teams, each under the responsibility of a medical doctor, who was also trained in verbal autopsies.

### Questionnaire and data processing

We used the standardized verbal autopsy questionnaires sponsored by WHO (version 2016 - 1.5.1). This version is optimized for automated computerized modelling using various algorithms (such as InterVA, Tariff, and InSilicoVA) [23]. The verbal autopsy guide, available in English was translated into French, and completed with some questions on reasons for death registration and sources of information on death reporting [30]. It was then loaded onto Android tablets as an XLSform file and filled in through the Open Data Kit (ODK) application, to take advantage of the numerous skip patterns in interviews. Causes of death were assigned using the “InterVA5" package of the R software [31], which is aligned with international standards set by the World Health Organization for verbal autopsies. InterVA is a freely available model (http://www.interva.net) that uses Bayes’ theorem to associate the probability of an event (a specific cause of death) occurring given a certain circumstance with the unconditional probability of that event and the conditional probability of that circumstance given the event [32]. The model produces values for the likelihood of each cause, based on the symptoms that are identified as present at the time of the interview, combined with a set of conditional probabilities developed by experts based on a variety of sources (including physician data). For each death, InterVA will sum the highest likelihoods to obtain the fraction of deaths attributable to the different causes at the population level [23]. InterVA provides estimates for the 3 most probable causes for each death if these likelihoods are above a certain threshold, otherwise, the cause is considered as undetermined. Causes of death are organized using the WHO short list, which identifies 62 causes of death.

The InterVA-5 model, applied to the 2016 WHO questionnaire, also identifies the circumstances of death, using a categorization called COMCAT (Circumstances Of Mortality CATegories) [33, 34]. This categorization refers to the limiting factors that may have hindered seeking and obtaining care at the time of death. It includes a dimension relating to the recognition of the importance of seeking care (based on questions relating to doubts about care and the use of traditional medicine), a dimension relating to accessibility (based on questions relating to the cost of care and transport), and a dimension relating to the quality of care (based on questions relating to the difficulties listed below). InterVA-5 calculates the likelihood that each death is attributable to one of the following seven categories: (1) *Traditions* (cultural factors that influenced care-seeking or disease progression to death), (2) *Emergencies* (a sudden, urgent or unexpected situation that prevented life-saving actions), (3) *Health system* (system deficiencies in terms of admission or treatment), (4)* Inevitability* (deaths that occurred in circumstances that could not have been reasonably avoided (e.g., terminally ill)), (5) *Recognition* (lack of awareness of the importance of the disease, or lack of knowledge of the symptoms of the disease), (6) *Resources* (inability to use or mobilize resources for treatment or transportation), and finally, (7) *Multiple factors* (a combination of the various factors above with no single factor predominating).

## Results

### Recruitment process

**Fig. 1:**
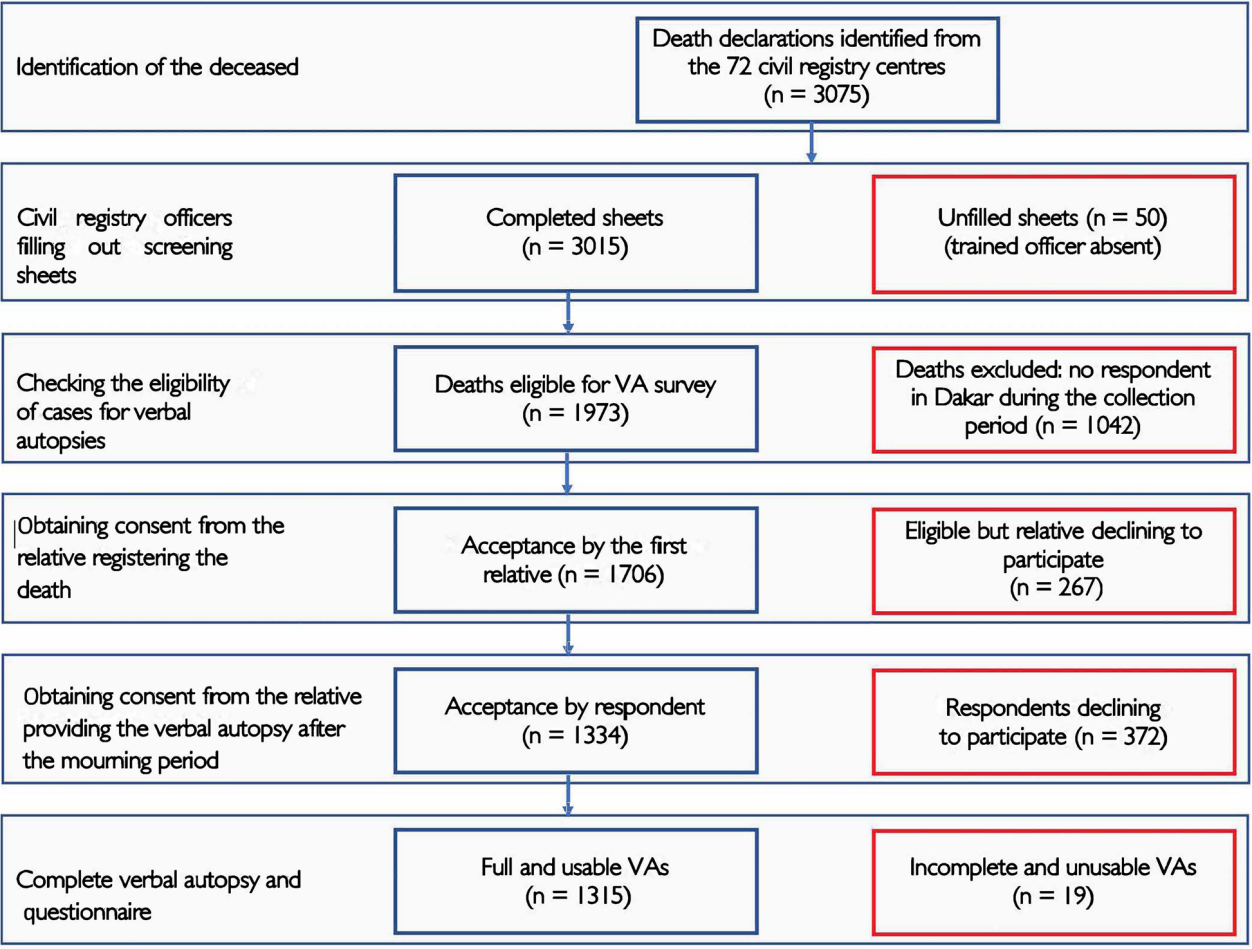
Flowchart summarizing the selection of deaths eligible for a verbal autopsy, Dakar pilot study, 2019

Between December 2018 and February 2019, 3,075 deaths were registered in registration offices of the Dakar region. A proportion of 55.0% of deaths was registered on the day of occurrence and 29.7% were registered the following day. 98.0% of the deaths (3015) were notified when a civil registrar trained in the survey procedures was available to collect the consent of the relative and verify the eligibility for verbal autopsy. Of these 3015 deaths, 1042 cases, or 34.6%, were not eligible for verbal autopsy because of the absence of a relative in the Dakar region during the collection period, because the deceased did not live in the region, or because it was a stillbirth. This high percentage can be explained by the fact that the structure of health care provision is highly concentrated in the Dakar region, especially for serious cases and those requiring medical specialties not available in other regions. Hence a total of 1973 deaths were eligible for this survey. In the registration offices, 86.5% (1706) of the relatives agreed to participate in the study and to share their contact details or those of a relative of the deceased. The investigators were then able to recontact 1334 respondents (78.2%) who in turn agreed again to participate in the study. Of the 1334 verbal autopsies performed, 1315 (98.6%) were complete and amenable to interpretation by statistical methods. In summary, of the 3075 deaths notified to registration offices during the study period, 1315 were fully investigated, or 42.8%. Figure 1 illustrates the selection process. Verbal autopsies were performed between 7 and 21 days after the date of declaration of death to one of the registration offices. The respondent for the verbal autopsy was primarily a parent of the deceased (33.8%), another family member (32.6%), a child of the deceased (20.8%), a spouse (9.5%), or other relatives, such as friends or health care personnel (3.3%).

### Age and sex distribution

Stillbirths were not eligible for a verbal autopsy. They are normally recorded in dedicated registers, or identifiable via a mention in the main register. However, some stillbirths were identified in the sample at the time of analysis, after being reported as live births in death registers. Based on four questions about signs of life (e.g. “Did the baby cry at least once?"), 127 stillbirths could be identified, representing 9.7% of the total number of deaths for which a verbal autopsy was administrated. This suggests that a significant proportion of registered deaths should not be included in the classical mortality indicators such as crude death rate or life expectancy, but should be subject to specific treatments reserved for measuring stillbirth rates. Our study was not intended to identify neonatal deaths reported to vital statistics as stillbirths, so it is difficult to predict the effect of misclassifications on mortality estimates derived from death registration data.

After excluding stillbirths, the sample consisted of 14.8% of infant deaths (11.3% were neonatal deaths and 3.5% were post-neonatal deaths), 1.7% of deaths that occurred between the 1st and 5th birthdays, 1.8% between 5 and 14 years of age, 34.7% between 15 and 59 years of age, and finally, 47.1% that occurred above age 60.

**Fig. 2:**
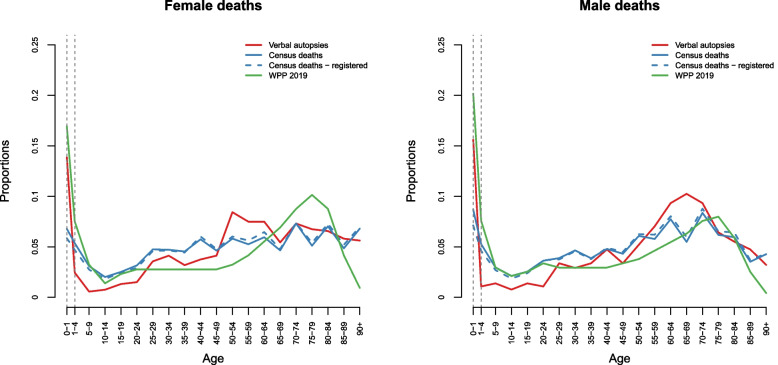
Age distribution of deaths in the 2019 verbal autopsy survey and the 2013 census, Dakar region, alongside estimates from the World Population Prospects 2019 for Senegal

Figure 2 compares the age distribution of deaths to that reported in the 2013 census for the Dakar region. The distribution based solely on deaths for which the household head indicated that they had been registered to the CRVS system is in dashed lines. This figure also displays the age distribution of deaths for the national population, as estimated by the United Nations for the period 2015-2020 [11]. These age structures differ substantially. The proportion of infant deaths in the verbal autopsy study (14.8%) is much higher than that observed in the census (7.8% of all deaths and 6.6% of registered deaths). However, it is close to the share of infant deaths expected at the national level from UN estimates (18.6%)^1^. Children who died at a very young age may have been omitted from the census, as has been observed in rural areas of Senegal [35]. It also is possible that age errors in the census caused some infant deaths to be transferred to older ages; typically, children who died before their first birthday may have been reported as having died at one year of age. The proportion of deaths under 5 years of age (16.5%) calculated in the verbal autopsy survey is more consistent with that calculated in the census (13.1%). The verbal autopsy survey also had a very high proportion of neonatal deaths, well above the expected level. According to the UN IGME [12], the neonatal mortality rate in 2019 throughout Senegal was estimated to be 22 deaths per thousand live births, while the under-five mortality rate was estimated to be 45 per thousand live births, thus a proportion of 0.49 of neonatal deaths, compared to 0.68 in our survey. This could reflect a more systematic registration of deaths occurring directly after delivery, compared to deaths of older children. In Dakar, some civil registration offices are adjacent to or integrated with hospitals, and medical staff may have encouraged parents to register the death.

Among adults, there is also some variation between age structures, with fewer deaths between the ages of 15 and 45 than expected from the census, compared to deaths in adults aged 45 years and older. The age structure of death in United Nations estimates shows a concentration of deaths in the older age groups (>65). This partly reflects the higher proportion of the population above age 60 in other parts of the country. Because of the lack of an appropriate age structure of death that could serve as a reference, we chose not to re-weight the data and retained the structure observed in the sample. We present results for neonatal deaths, deaths among children and adolescents (aged 1 month to 15 years), adults (15-64 years) and elderly (65+).

### Place of death and health care seeking

44.1% of deaths occurred at home (Table 1) and the proportion of home deaths increased with age; from 10.4% for newborns, it reached 61.1% in the elderly. Deaths occurring in health facilities represented 50.0% of all deaths, while those occurring on the way to a health care facility or in other places (e.g. in a school or work setting, or on public roads) represented about 6% of all deaths.

**Table 1:** Place of death, health care seeking and circumstances of deaths analyzed with verbal autopsies (% in columns) (*n*=1188), Dakar pilot study, 2019

	Neonates	Children/adolescents	Adults	Elderly	All
	(<1 month)	(1m-14y)	15-64y	65+y	
**Place of death**					
Hospital	74.6	51.8	44.8	31.2	43.4
Other health care facility	13.4	4.8	7.8	3.7	6.6
On the way to a health care facility	0.7	4.8	6.2	2.4	4.0
Home	10.4	34.9	39.2	61.1	44.1
Other (public road, etc.)	0.7	3.6	1.9	1.5	1.8
**Doubts about the need for medical care in the final days**					
Yes	11.9	21.7	22.2	19.4	19.9
No	84.3	78.3	76.4	79.3	78.5
Don’t know	3.7	0.0	1.4	1.3	1.5
**Visit to a health facility in the final days**					
Yes	73.9	57.8	63.2	48.9	58.5
No	25.4	41.0	35.5	50.0	40.3
Don’t know	0.7	1.2	1.4	1.1	1.2
**Circumstances Of Mortality CATegories (COMCATs)**					
Traditions	0.7	7.2	7.2	5.5	5.8
Emergency	40.3	25.3	14.0	4.4	14.1
Health systems	21.6	12.0	9.2	4.1	8.8
Inevitable	28.4	2.4	28.1	56.1	37.1
Recognition	0.7	33.7	25.1	19.2	20.7
Resources	4.5	16.9	13.6	7.9	10.6
Multiple	3.7	2.4	2.7	2.8	2.9
Number of deaths	134	83	513	458	1, 188

For 78.5% of deaths, the family had no doubts about the need for medical care in the last days before death. The importance of seeking care was recognized for all age groups in comparable proportions (76-84%) (Table 1). However, for only 58.5% of the deaths, a health facility was visited in the final days. The proportion of cases that received medical assistance decreased gradually with age, from 73.9% among newborns to 48.9% among the elderly. Among those for which the need for care was established, the proportion of individuals who did not receive medical assistance amounts to 43.8% overall and varies from 39.3% among newborns to 53.1% among the elderly. Hence the lower use of end-of-life care among the elderly is not due to lower recognition of the critical nature of their health condition, but rather to the fact that their forthcoming death was perceived as inevitable or to other factors hindering access to care.

As the Dakar region is well served by health facilities, only 7.5% of respondents stated that it took more than two hours to get to the nearest hospital or health facility from the deceased’s home. The barriers to accessing care are therefore of a different nature than physical accessibility alone. Among those who sought medical assistance, one person in five (21.0%) encountered difficulties in being admitted to a hospital or health centre a few days before the death (Table 2). One person in four (24.9%) also experienced difficulties with the way they were treated by health care providers (difficulties with medical treatment, procedures, interpersonal attitude, respect, dignity, etc). Finally, for 16.0% of cases, difficulties were encountered in obtaining medication or diagnostic tests. In total, more than one-third of those for whom medical assistance was sought before death experienced at least one type of difficulty (37.7%). Difficulties in accessing care were not more frequent among the elderly, and therefore cannot explain their lower use of care and the higher frequency of death at home observed among them.

**Table 2:** Difficulties encountered in the final days for those who sought care (% in columns) (*n*=695), Dakar pilot study, 2019

	Neonates	Children/adolescents	Adults	Elderly	All
	(<1 month)	(1m-14y)	15-64y	65+y	
**Difficulties in being admitted to a hospital or health center**					
Yes	22.2	12.5	24.1	17.9	21.0
No	77.8	87.5	74.7	82.1	78.4
Don’t know	0.0	0.0	1.2	0.0	0.6
**Difficulties with the way he/she was treated in the hospital or health facility**					
Yes	31.3	14.6	25.3	23.7	24.9
No	68.7	85.4	72.5	76.3	74.1
Don’t know	0.0	0.0	2.2	0.0	1.0
**Difficulties in obtaining medication or diagnostic tests at the hospital or health facility**					
Yes	16.2	12.5	19.1	12.1	16.0
No	82.8	87.5	78.7	87.5	82.7
Don’t know	1.0	0.0	2.2	0.4	1.3
Number of deaths	99	48	324	224	695

### Circumstances of mortality

Table 1 details the circumstances of deaths for each of the four age groups. Among neonates, 40.3% could be attributed to an emergency situation, 28.4% were considered unavoidable, and 21.6% were attributed to health system deficiencies. Lack of knowledge of symptoms or lack of awareness of the severity of the disease played a much larger role in children aged between one month and 15 years: 33.7% of deaths were associated with this category, compared with less than one percent of neonatal deaths. About a quarter of child deaths (25.3%) were caused by sudden, urgent, or unexpected problems. Next, the inability to find or mobilize resources and problems with medical care together caused about one-quarter of deaths (16.9 and 12.0%, respectively). Among adults aged 15-64 years, the three most frequent types of circumstances were, in decreasing order, the inevitability of death (28.1%), lack of knowledge of the disease or its severity (25.1%), emergencies (14.0%) and lack of resources (13.6%). Finally, among the elderly, cases of death deemed inevitable represented more than half of the sample (56.1%), followed by lack of knowledge of the disease or its severity (19.2%) and lack of resources (7.8%).

### Leading causes of death

Tables 3, 4, 5 and 6 display the cause-specific mortality fractions (CSMF), expressed in percentages, for each age group. For neonatal deaths or those occurring in childhood and adolescence, the CSMF values are presented only when they are greater than 5%. For adults and the elderly, the number of deaths in our sample is higher, and cause-specific mortality fractions are presented when greater than 2%. Undetermined causes are also presented to assess the ability of the questionnaire and algorithm to establish probable causes of death.

#### Neonatal deaths

For the 134 neonatal deaths, the four leading causes of death were, in descending order, prematurity, accounting for 54.8% of deaths, birth asphyxia (24.2%), congenital malformations (10.5%), and neonatal sepsis (5.2%) (Table 3). Together, these four causes accounted for 94.6% of neonatal deaths. Only 2.4% of deaths had an undetermined cause, while 3.0% of deaths had other defined causes (neonatal pneumonia, other neonatal disorders). There was little variation by sex in the cause of death distribution in neonates, but boys were overrepresented in the sample (1.35 newborn male deaths for every 1 female death).

**Table 3:** Cause-specific mortality fractions (%) among neonates (*n*=134), Dakar pilot study, 2019

	Total	Boys	Girls
Prematurity	54.8	55.8	53.5
Birth asphyxia and birth trauma	24.2	23.4	25.2
Congenital anomalies	10.4	11.7	8.8
Neonatal sepsis	5.2	5.2	5.2
Other causes	3.0	2.6	3.5
Undertermined	2.4	1.3	3.8
Number of deaths	134	77	57

#### Deaths in children and adolescents

Between one month and 15 years of age, the distribution of causes of death is less concentrated. 35.9% of deaths are attributed to causes whose CSMF is lower than 5%. Meningitis and encephalitis together form the leading category, causing 23.3% of deaths at these ages (Table 4). This large proportion should be put into perspective because the survey covers deaths that occurred between December and February, during the dry season that is conducive to the development of meningitis [36]. Severe malnutrition is the second category, causing 16.2% of deaths for both sexes combined. More unexpectedly, 6.4% of deaths are attributed to HIV/AIDS, while HIV prevalence remains very low in the Dakar region (0.3% of the adult population according to the 2017 DHS survey [28]).

**Table 4:** Cause-specific mortality fractions (%) among children and adolescents (*n*=83), Dakar pilot study, 2019

	Total	Boys	Girls
Meningitis and encephalitis	23.3	19.5	28.0
Severe malnutrition	16.2	15.6	17.0
Acute resp infect (incl pneumonia)	7,1	5,5	9.1
HIV/AIDS related death	6.4	5.9	7.1
Other and unspecified infect diseases	4,0	7,1	0,0
Other causes of death	31.9	32.6	31.1
Undetermined	11.0	13.7	7.7
Number of deaths	83	46	37

#### Deaths in adults aged 15-64

The leading causes of death among adults are presented in Table 5. 53,2% of these deaths were among men. The leading cause of death was stroke (12.9% of deaths), which can be associated with other unspecified heart diseases (8.7%) and acute cardiac disease (5.6%) accounting together for more than one-quarter of adult deaths in this age group (27.2%). Cancers together form the second major category of causes of death, with digestive neoplasms (8.1%) in the first place, followed by respiratory neoplasms (3.7%), reproductive neoplasms (3.7%), and other cancers (2.6% of deaths). Overall, the fraction of deaths in adults caused by cancers is 20.2%, just after cardiovascular diseases. Diabetes caused 3.0% of deaths. Again, somewhat surprisingly, 6.7% of deaths are associated with HIV/AIDS, and this proportion is 8.8% for women, compared to 4.9% for men. This appears to be a significant overestimate when considering HIV prevalence. A more detailed examination is needed to better understand why these deaths were attributed by the algorithm to HIV/AIDS and what their true causes might be. Even without these HIV/AIDS deaths, communicable diseases still account for a significant proportion of deaths among adults. Pulmonary tuberculosis, acute respiratory infections, meningitis and encephalitis, and diarrheal diseases are the most important, and together they cause 14.8% of deaths. Two other categories of causes of death affect men and women differentially; road traffic accidents, which cause 4.2% of male deaths, and maternal disorders (7.3% of female deaths, mainly due to pregnancy-induced hypertension and obstetric haemorrhage).

**Table 5:** Cause-specific mortality fractions (%) among adults aged 15-64 (*n*=513), Dakar pilot study, 2019

	Total	Men	Women
Stroke	12.9	9.2	17.2
Other and unspecified cardiac diseases	8.7	6.2	11.5
Digestive neoplasms	8.1	10.2	5.7
Pulmonary tuberculosis	7.4	10.9	3.4
HIV/AIDS related death	6.7	4.9	8.8
Acute cardiac disease	5.6	7.2	3.7
Acute resp infect incl pneumonia	5.0	5.3	4.6
Respiratory neoplasms	3.7	5.3	1.9
Reproductive neoplasms	3.7	0.3	7.6
Pregnancy-related deaths	3.4	0.0	7.3
Diabetes mellitus	3.0	3.3	2.7
Other and unspecified neoplasms	2.6	3.5	1.6
Renal failure	2.3	2.2	2.5
Road traffic accident	2.2	4.2	0.0
Diarrhoeal diseases	1.4	2.1	0.6
Other causes	16.7	16.1	17.4
Undetermined	9.8	9.0	10.7
Number of deaths	513	273	240

#### Deaths in adults aged 65 and above

The leading cause of death among adults aged 65 and above was again stroke (25.1% of deaths) (Table 6). It may be associated with unspecified heart disease, the second leading cause of death, accounting for 12.6% of cases, and acute heart disease (6.9%). Together, these cardiovascular diseases cause more than two-fifths of deaths in the elderly (44.6%). Diabetes mellitus ranks third (8.3%). The fifth leading cause of death is pulmonary tuberculosis (4.4%), which causes significantly more male than female deaths (6.1 versus 2.3%). Digestive neoplasms come next, again the most common cancer category in our sample of deaths. Overall, cancers caused 12.4% of deaths in this age group. The proportion of deaths for which verbal autopsies could not determine a cause was 12.9%, which is the highest proportion of the four age groups.

**Table 6:** Cause-specific mortality fractions (%) among adults aged 65 and above (*n*=458), Dakar pilot study, 2019

	Total	Men	Women
Stroke	25.1	23.1	27.7
Other and unspecified cardiac diseases	12.6	15.5	8.9
Diabetes mellitus	8.3	9	7.4
Acute cardiac disease	6.9	6.8	7.1
Pulmonary tuberculosis	4.4	6.1	2.3
Digestive neoplasms	3.9	4.6	2.9
Other and unspecified neoplasms	3.5	5.1	1.5
Acute resp infect incl pneumonia	3.1	3.0	3.3
Reproductive neoplasms	2.6	0.9	4.9
Renal failure	2.3	2.2	2.4
HIV/AIDS related death	2.1	2.6	1.5
Respiratory neoplasms	2.1	3.3	0.5
Other causes	10.2	7.5	13.6
Undetermined	12.9	10.5	16.0
Number of deaths	458	258	200

## Discussion

In recent years, there has been renewed interest in improving birth and death registration in many low- and middle-income countries. Efforts have been made to assess the status of civil registration systems [1, 37], improve mechanisms for collecting causes of death [23, 38], revise legal frameworks, and strengthen civil registration administrations [39]. The Senegalese government is participating in this effort. It has taken several initiatives to support its civil registration system, such as creating the National Center for Civil Registration (CNEC) in 2004 (now called Directorate of Civil Registration (DEC)) and initiating the digitization of birth and death certificates. However, even in regions where the completeness of death registration is sufficient to produce reliable mortality indicators, the system does not allow for the tracking of causes of death, as these are not collected. Our study demonstrates that verbal autopsies could be integrated with the CRVS system in Dakar (and possibly other large urban areas) to fill such data gaps.

To be scaled up, this approach should address the numerous system-level challenges associated with this integration: de Savigny and colleagues (2017) advise that all the stakeholders and sub-systems of the CRVS system be considered, that verbal autopsies be integrated into the existing information flow and work process, that the VA results be connected with the death records, and that a series of technical constraints be taken into account [40]. We did not incorporate all these constraints in our pilot study because addressing them requires concerted action by different ministries (health, local governance, interior, justice) and partners. Our study does, however, demonstrate the feasibility of recruiting respondents for verbal autopsies through civil registration offices, and the plausibility of the estimates obtained. This is a rapid and standardized approach that provides estimates that are comparable with data from rural HDSS or other countries, at a relatively low cost because the notification of deaths is passive and relies on families and relatives.

A series of recommendations can be made based on this pilot study before wider implementation. First, a screening based on registered deaths cannot be exhaustive. A fraction of unreported deaths will not be included, and these deaths could systematically differ from those registered, introducing selection biases. The integration of verbal autopsies in death registration offices should therefore be limited to areas where death completeness is sufficiently high, such as in some cities of Senegal (Dakar, Saint-Louis, Ziguinchor, etc.). Further research is also required to design ways to correct for such biases, for example by re-weighting the data according to certain known characteristics associated with completeness (age, sex, neighbourhood of residence). Second, the cause-of-death structure is variable over time with a pattern that may change with the seasons of the year. This study was carried out over a limited period of the year, leading to the overrepresentation of some causes (e.g. meningitis and encephalitis). It is therefore recommended that a sample be taken on an annual basis, possibly including only a part of the deaths notified each month if budgetary constraints make it impossible to achieve exhaustiveness. Third, any verbal autopsy survey based on notifications made at vital registration centers should specify the eligibility criteria, acknowledging that these can significantly reduce inclusion rates. Typically, vital registration centers located near major hospitals collect a high number of deaths, but a larger fraction of these reports are represented by ineligible cases (persons not living in the area, stillbirths, etc.). Misclassification of stillbirths as neonatal deaths seems common; some identification questions based on signs of life should be routinely asked when neonatal deaths are being registered for a more accurate measurement of neonatal and stillbirth rates. Our study showed that stillbirths accounted for a large percentage of death registered, which contrasts with an earlier study conducted in four HDSS sites in Ethiopia, Ghana, Guinea-Bissau and Uganda, which found that only a very small fraction of stillbirths were registered (2.5%) [41]. Fourth, the full collaboration of the staff from registration offices is fundamental in this type of design. As the staff is in charge of screening eligible cases, insufficient training or motivation of the agents will lead to declines in inclusion rates and will compromise the quality of the data. When these conditions are met, relatives eligible for verbal autopsies are usually willing to participate in the survey, but they are not always the person designated initially as the respondent. Indeed, the verbal autopsy guide can only be administered to people who have been in close contact with the deceased, and who know the symptoms as well as the circumstances in which the person died. In our study, this person was often different from the one who declared the death at the civil registration office, so two levels of consent should be considered. In addition to refusals at civil registration offices, secondary refusals, made by targeted respondents who are contacted after a period of mourning, must be also anticipated. When administering verbal autopsy guides, capturing the complete information may require the participation of two to three people, especially for the deaths of adults or the elderly. Our experience is that children of the deceased, often responsible for driving the deceased to the hospital and assisting them during hospitalizations, have more control over the therapeutic journey, diagnosis, and treatment.

Beyond these methodological considerations, this survey conducted in the civil registration centers in the Dakar region sheds light on the cause-of-death structure prevailing in this urban area.

Among neonates, nine-tenths of the deaths in our sample were attributed to only three causes: prematurity, obstetric asphyxia, and congenital malformations. The vast majority of these deaths are preventable with targeted interventions: improvement of the nutritional status of mothers, promotion of contraception to space births, strengthening of pregnancy care structures (prenatal visits and assistance at delivery), reduction and screening for hypertension, early initiation of breastfeeding, etc. The distribution of neonatal deaths by cause in this study contrasts with WHO’s modelled estimates for Senegal. According to WHO, prematurity caused 27% of neonatal deaths in 2019, and neonatal sepsis 21% (against 54.8% and 5.2% in our study). No deaths related to acute respiratory infections were identified in the sample, although this cause accounted for 7% of deaths in the country according to WHO [42].

Beyond the first month, meningitis and encephalitis, severe malnutrition, acute respiratory infections, HIV/AIDS and other infectious diseases were the leading causes of death. Here again, effective and inexpensive interventions can reduce this mortality through the promotion of exclusive breastfeeding, vaccination, vitamin A supplementation, immunization, management of pneumonia, etc. The cause-of-death distribution in children and adolescents also differed from the WHO model-based estimates. According to WHO, less than 1% of deaths were caused by HIV/AIDS in Senegal among children aged less than 15 [42], against 6.4% in our sample (excluding neonates). The share of deaths caused by HIV/AIDS has likely been overestimated in verbal autopsies; these deaths may instead have been caused by tuberculosis or other conditions. This points to a limitation of the WHO questionnaire and the probabilistic model of attribution of causes applied to the Senegalese context, at least for children. Malaria was not among the leading causes of death in children (the fraction of malaria deaths is estimated at only 0.5% in this age group), while it caused 4,9% of deaths under the age of 15 in Senegal according to WHO [42]. This discrepancy is expected, however, as Dakar has a lower malaria burden than the rest of the country, thanks to the scale-up of interventions over the last decades [43].

After the 15th birthday, cardiovascular diseases caused 27.2% of adult deaths between the ages of 15 and 64 in the sample and 44.6% over the age of 65. This is higher than WHO estimates, according to which the cause-specific mortality fraction of cardiovascular diseases was 21.6% in the age group 15-59 and 33.1% above age 60. In our study, neoplasms also caused 20.2% of deaths between 15 and 64 years of age, and 12.4% over 65 years of age, in line with WHO estimates (respectively 20.1% in the age group 15-59 and 11.9% in adults aged 60 and above). A multitude of genetic, physiological, environmental and behavioral factors are at work in causing these deaths. However, a small group of modifiable risk factors have been identified and can be targeted by prevention policies: smoking, lack of physical activity, excessive alcohol consumption, and a diet too rich in sugar, salt, and saturated fatty acids. A study conducted in 2015 in Dakar estimated the prevalence of hypertension at 24.7% among adults aged 20 to 89 years, a level close to those estimated in some high-income countries such as the United States. Only 28.4% of hypertensive individuals were aware of their condition. According to the same study, 19.2% of adults in Dakar were overweight and 9.7% were obese [21].

## Conclusion

This study demonstrates the value of conducting regular studies based on verbal autopsies of deaths reported in civil registration offices to monitor the epidemiological transition in urban Senegal. The Dakar region is already at an advanced stage of this transition, as the cause-of-death hierarchy is largely dominated by non-communicable diseases above age 15.

## Data Availability

The anonymized verbal autopsy data are available upon request to the Agence Nationale de la Statistique et de la Démographie (ANSD).

## Footnotes

1 The UN WPP estimates do not allow us to separate infant deaths from child deaths, but the share of infant deaths among under-five deaths was taken from UN IGME for the year 2017 [12].
